# DDB2 represses epithelial-to-mesenchymal transition and sensitizes pancreatic ductal adenocarcinoma cells to chemotherapy

**DOI:** 10.3389/fonc.2022.1052163

**Published:** 2022-12-08

**Authors:** Julie Dardare, Andréa Witz, Margaux Betz, Aurélie Francois, Morgane Meras, Laureline Lamy, Aurélien Lambert, Stéphanie Grandemange, Marie Husson, Marie Rouyer, Jessica Demange, Jean-Louis Merlin, Alexandre Harlé, Pauline Gilson

**Affiliations:** ^1^ Université de Lorraine, Centre National de la Recherche Scientifique (CNRS), Unité Mixte de Recherche (UMR) 7039 Centre de Recherche en Automatique de Nancy (CRAN), Nancy, France; ^2^ Service de Biopathologie, Institut de Cancérologie de Lorraine, Vandoeuvre-les-Nancy, France

**Keywords:** damage specific DNA binding protein 2, pancreatic ductal adenocarcinoma, epithelial-to-mesenchymal transition, chemosensitivity, biomarker, prognosis

## Abstract

**Introduction:**

Damage specific DNA binding protein 2 (DDB2) is an UV-indiced DNA damage recognition factor and regulator of cancer development and progression. DDB2 has dual roles in several cancers, either as an oncogene or as a tumor suppressor gene, depending on cancer localization. Here, we investigated the unresolved role of DDB2 in pancreatic ductal adenocarcinoma (PDAC).

**Methods:**

The expression level of DDB2 in pancreatic cancer tissues and its correlation with patient survival were evaluated using publicly available data. Two PDAC cell models with CRISPR-modified DDB2 expression were developed: DDB2 was repressed in DDB2-high T3M4 cells (T3M4 DDB2-low) while DDB2 was overexpressed in DDB2-low Capan-2 cells (Capan-2 DDB2-high). Immunofluorescence and qPCR assays were used to investigate epithelial-to-mesenchymal transition (EMT) in these models. Migration and invasion properties of the cells were also determined using wound healing and transwell assays. Sensitivity to 5-fluorouracil (5-FU), oxaliplatin, irinotecan and gemcitabine were finally investigated by crystal violet assays.

**Results:**

DDB2 expression level was reduced in PDAC tissues compared to normal ones and DDB2-low levels were correlated to shorter disease-free survival in PDAC patients. DDB2 overexpression increased expression of E-cadherin epithelial marker, and decreased levels of N-cadherin mesenchymal marker. Conversely, we observed opposite effects in DDB2 repression and enhanced transcription of SNAIL, ZEB1, and TWIST EMT transcription factors (EMT-TFs). Study of migration and invasion revealed that these properties were negatively correlated with DDB2 expression in both cell models. DDB2 overexpression sensitized cells to 5-fluorouracil, oxaliplatin and gemcitabine.

**Conclusion:**

Our study highlights the potential tumor suppressive effects of DDB2 on PDAC progression. DDB2 could thus represent a promising therapeutic target or biomarker for defining prognosis and predicting chemotherapy response in patients with PDAC.

## Introduction

Pancreatic cancer is one of the most lethal cancers with a 5-year relative survival rates about 9% (1). A total of 498,773 estimated new cases were diagnosed worldwide in 2020 associated with more than 466,000 deaths related (2). Pancreatic ductal adenocarcinoma (PDAC), which represents about 90% of pancreatic cancer, is an aggressive disease characterized by high tumor heterogeneity, strong metastatic potential and resistance to chemotherapy. PDAC is often diagnosed at late stage; only 20% of patients can be eligible to surgical resection which remains today the only potentially curative treatment (3). Current standard of care for patients with locally advanced disease or distant metastases consists of either gemcitabine or FOLFIRINOX (5-fluorouracil, leucovorin, irinotecan, oxaliplatin) (4). Recently, FDA approved Olaparib for maintenance therapy in patients with germline *BRCA1* or *BRCA2* mutations (5). Pembrolizumab is also approved in patients with metastatic PDAC harboring defects in mismatch repair or microsatellite instability (6, 7). Despite these recent advances targeted therapies still have a limiting role in the management of PDAC, highlighting the urgent need to identify new therapeutic targets.

*DDB2* (Damage Specific DNA Binding Protein 2) gene, mapped to chromosome 11p11.2, encodes a 48 kDa protein originally identified to be implied in the recognition of ultraviolet-induced DNA damage and the initiation of nucleotide excision repair (NER) process (8). Beyond its well established functions in DNA repair, DDB2 appears as a multifunctional protein implied in the development and the progression of various cancers with both anti-oncogenic and pro-oncogenic roles (9). On one side, DDB2 was shown to promote the proliferation of breast cancer cells (10) as well as the migration and invasion properties of gastric cancer cells (11). On the other side, DDB2 has showed antiproliferative activities in ovarian (12) and prostate cancer (13). In addition, DDB2 reduces motility and invasiveness of breast cancer cells and represses epithelial-to-mesenchymal transition (EMT) in colon cancer (14) and in oral/head and neck squamous cell carcinoma (HNSCC) (15).

EMT has been largely described as a major driver of PDAC progression and metastasis (16). EMT is a dynamic process during which cells undergo molecular changes that switch their epithelial phenotype toward a mesenchymal phenotype. EMT is triggered through different signaling pathways including transforming growth factor β (TGF-β), Notch, Wnt/β-catenin, epidermal growth factor (EGF), fibroblast growth factor (FGF), and platelet-derived growth factor (PDGF) (17–20). These signaling pathways lead to the activation of EMT transcription factors (EMT-TFs) including Snail1, Snail2, Zeb1, Zeb2, Twist1 and Twist2 (21–24). Altogether, these EMT-TFs lead to the repression of the E-cadherin epithelial protein, which is a crucial step of EMT. EMT-TFs also induce the expression of different mesenchymal proteins such as N-cadherin, vimentin, smooth muscle actin or fibronectin (25).

In this study, we found that DDB2 expression decreases during PDAC progression and is associated with poor outcomes. We show that DDB2 can repress EMT, migration and invasion in PDAC cell models. We also found that DDB2 can sensitize PDAC cells to chemotherapy. Taking together these findings bring new insights of the promising use of DDB2 as a potent biomarker to define prognosis and to predict chemotherapy response of PDAC patients.

## Materials and methods

### DDB2 expression analyses in publicly available data

The relationship between the mRNA DDB2 expression level and the prognosis of PDAC patients was analyzed using the online database Kaplan-Meier-plotter (www.kmplot.com). Overall survival was performed based on a cohort of 177 patients and disease-free survival was established using a cohort of 69 patients. Analyses of DDB2 proteomic expression in tumors from patients were performed using UALCAN (http://ualcan.path.uab.edu), a user-friendly interactive web resource for analyzing OMICS cancer data (26). Transcriptomic expression analysis was performed using a PDAC cohort of 178 patients from The Cancer Genome Atlas (TCGA) and proteomic expression analyses were performed using a PDAC cohort of 137 patients from the Clinical Proteomic Tumor Analysis Consortium (CPTAC).

### Cell lines

The Capan-2 (RRID: CVCL_0026) PDAC cell line and the hTERT-HPNE (RRID: CVCL_C466) pancreatic cell line were obtained from the American Type Culture Collection (ATCC) (Manassas, VA, USA). The T3M4 (RRID: CVCL_4056) PDAC cell line was kindly donated by Professor Jens Werner from University of Heidelberg, Germany (27). MDA-MB-231 (RRID: CVCL_0062), MCF-7 (RRID: CVCL_0031) and T47D (RRID : CVCL_0553) human breast cancer cell lines were generously provided by Professor Philippe Becuwe from Lorraine University, UMR CNRS 7039 CRAN, France (28) and were used as DDB2-high and low controls respectively. Cell lines were cultured in humidified incubator with 5% CO_2_ at 37°C in antibiotic-free RPMI 1640 (Gibco, Carlsbad, CA, USA) supplemented with 10% heat inactivated fetal bovine serum (FBS) and 2 mM L-glutamine (Sigma-Aldrich Corp., St. Louis, MO, USA). Cells were periodically tested for *Mycoplasma* contamination using the VenorH GeM Mycoplasma Detection Kit (Minerva Biolabs GmbH, Berlin, Germany). All experiments were performed within 3–10 passages after thawing the cells.

### Transfection

Approximately 1.5x10^5^ of T3M4 cells and 2.5x10^5^ of Capan-2 cells were seeded into a 6-well plate and incubated for 24 hours. Then, cells were transfected using jetPRIME Transfection Reagent (Polyplus transfection, Illkirch, France). T3M4 cells were transfected with DDB2 CRISPR/Cas9 KO plasmid (cat# sc-401686, Santa Cruz Biotechnology, Dallas, TX, USA) and selected with 1.5 µg/ml of puromycin or with control CRISPR/Cas9 plasmid (cat# sc-418922, Santa Cruz Biotechnology). Capan-2 cells were transfected with DDB2 CRISPR Activation plasmid (cat# sc-401686-ACT, Santa Cruz Biotechnology) and selected with 2µg/ml of puromycin, 1µg/ml of blasticidin and 200µg/ml of hygromycin or with control CRISPR activation plasmid (cat# sc-437275, Santa Cruz Biotechnology).

### Quantitative reverse-transcription PCR

Total RNA was extracted using RNeasy Mini Kit (Qiagen, Hilden, Germany) according to the manufacturer’s instructions. RNA was quantified using InvitrogenTM Qubit RNA HS Assay Kit (ThermoFisher Scientific, Inc. Waltham, MA, USA). cDNA was synthetized using the iScriptTM cDNA Synthesis Kit (Bio-Rad, Hercules, CA, USA) starting from 1µg total RNA. Quantitative PCR was performed in triplicate on the LighCycler^®^ 480 (Roche, Basel, Switzerland) using LightCycler^®^ 480 SYBR Green I Master kit. Primers were purchased from Eurogentec (Seraing, Belgium) and sequences are provided in **Table 1**. Results were normalized with endogenous β-Actin, using the standard ΔΔCt method.

**Table 1:** Sequences of primers used for RT-qPCR.

Gene	Forward sequence	Reverse sequence
**β-Actin**	AGAGCTACGAGCTGCCTGAC	AGCACTGTGTTGGCGTACAG
**DDB2**	CTCCTCAATGGAGGGAACAA	GTCACCACCATTCGGCTACT
**SNAIL**	TTCAACTGCAAATACTGCAACAAG	CGTGTGGCTTCGGATGTG
**ZEB1**	GATGATGAATGCGAGTCAGATGC	ACAGCAGTGTCTTGTTGTTGT
**TWIST**	CCCAAAAAGAAAGCGCCCAA	TCACGTCAGGCCAATGACAC
**E-cadherin**	GAACAGCACGTACACAGCCCT	GCAGAAGTGTCCCTGTTCCAG
**N-cadherin**	GACGGTTCGCCATCCAGAC	TCGATTGGTTTGACCACGG
**Bcl-2**	ATGTGTGTGGAGAGCGTCAA	ACAGTTCCACAAAGGCATCC

### Western immunoblotting

Cells were lysed using RIPA lysis buffer (Merck, Darmstadt, Germany) containing phenylmethylsulfonyl fluoride (PMSF) (Sigma-Aldrich Corp.) Lysates were centrifuged at 15,000 g for 20 minutes at 4°C and supernatants were stored at -80°C. Proteins were quantified using the DCTM Protein Assay Kit (Bio-Rad). Equal amounts of proteins (50 µg) were separated by 12.5% SDS-PAGE and electro-transferred to polyvinylidene difluoride membranes (Bio-Rad). Then, membranes were blocked with a solution of 0.1% Tween 20/Tris Buffered Saline (TBS) containing 5% non-fat milk for 1 hour at room temperature under agitation. The DDB2 (cat# ab181136, Abcam, Cambridge, United Kingdom) and α-Tubulin (cat# sc-23948, Santa Cruz Biotechnology) antibodies were diluted at 1:1,000, the E-cadherin (cat# 562869, BD Biosciences, Franklin Lakes, NJ, USA) and N-cadherin (cat# 14215S, Cell Signaling, Danvers, MA, USA) antibodies were diluted at 1:500. Primary antibodies were incubated overnight at 4°C. Secondary anti-rabbit HRP-linked (cat# 7074S, Cell Signaling) and anti-mouse HRP-linked (cat# 7076S, Cell Signaling) antibodies were applied for 1 hour at room temperature. Targeted proteins were detected using the Clarity Western ECL kit (Bio-Rad) and visualized with AzureC600 Camera (Azure Bio-systems, Dublin, CA, USA).

### Cell proliferation assay

A total of 1x10^4^ T3M4 and 1x10^4^ Capan-2 cells were seeded into a 12-well culture plate. Cells were incubated in culture medium for 192 hours and number of cells was determined every 24 hours. Cell population doubling time was estimated using the following formula:


Doubling time=t∗ln(2)/(ln(Nt)-ln(N0))

Where Nt corresponds to cell number at time t and N0 the cell number at the beginning of the experiment.

### Immunocytochemical analyses

Approximately 5x10^3^ T3M4 and 5x10^3^ Capan-2 cells were incubated on coverslips overnight. Cells were fixed with 4% paraformaldehyde/2% sucrose, then cells were permeabilized with PBS containing 0.2% Triton-X-100. Coverslips were blocked with PBS containing 3% BSA for 1 hour at room temperature. The rabbit anti E-cadherin (cat# MA5-14458, Invitrogen, 1:100) and mouse anti N-cadherin (cat# 14215S, Cell Signaling, 1:25) antibodies were incubated overnight at 4°C. Cells were washed with PBS following by 1 hour incubation with anti-rabbit Atto 550 secondary antibody (cat# 43328, Merck, 1:400) or anti-mouse CF™488A secondary antibody (cat# SAB4600388, Merck, 1:400). Coverslips were mounted onto glass slides using Vectashield Antifade mounting media counterstained with DAPI (Vector Laboratories, Burlingam, USA). Images were acquired using Olympus AX70 microscope (Olympus, Tokyo, Japan) using a 100x objective. All cells were excited at the same exposure time for each antibody.

### Wound healing assay

A total of 1x10^6^ cells was added into Ibidi^®^ Culture Insert chamber (Ibidi^®^, Gräfelf-ing, German). Inserts were removed after 24 hours (T3M4) or 48 hours (Capan-2) of incubation. Images of the wound were captured every 30 minutes for 16 hours using ImageX-press^®^ Micro Confocal system (Molecular Devices, San José, CA, USA). Wound closures were compared between 0 and 8 hours after insert removal, the area measurements were obtained using the ImageJ software.

### Transwell assay

Transwell assays were performed using Falcon^®^ Permeable Support with 0.8 µm PET membrane (cat#353097, Corning, Glendale, AZ, USA) and Corning^®^ BioCoat^®^ Growth Factor Reduced Matrigel^®^ Invasion Chambers with 8.0 µm PET membrane (cat#354483, Corning). Approximately 5x10^4^ T3M4 and 1x10^5^ Capan-2 cells were seeded into the upper chamber of inserts in serum-free medium and complete medium was added to the lower chamber. After 24 hours of incubation, non-invasive cells were removed by scrubbing the upper face of the membrane. Cells were fixed in 96% ethanol for 15 minutes and stained with 1% crystal violet (Sigma-Aldrich Corp.) for 15 minutes. Then inserts were washed and cells on the lower surface were examined using Olympus AX70 microscope using a 10x objective. Three random fields were photographed for counting cells. The percentage of invasion was determined as mean of invasive cells in inserts with Matrigel divided by the mean of migrative cells in inserts without Matrigel.

### Crystal violet assay

Approximately 5x10^3^ T3M4 and 2x10^4^ Capan-2 cells were seeded in each well of a 96-well plate. After overnight incubation, culture medium was replaced by dilution range of chemotherapies for 72 hours. The chemotherapies used were 5-Fluorouracil (5-FU), irinotecan, oxaliplatine and gemcitabine (Accord Healthcare, Lille, France). Then, cells were fixed with 70% ethanol for 10 minutes, followed by 0.2% crystal violet in 20% ethanol for 15 minutes. After three washes, 0.1% acetic acid in 50% ethanol was added in each well and optical density was measured at 540 nm using a microplate reader (Multiskan As-cent; Thermo Fisher Scientific). The half-maximal inhibitory concentration (IC50) values of each chemotherapy were determined by a non-linear regression using GraphPad Prism 9^®^ (GraphPad Software, La Jolla, CA, USA). All experiments were performed in triplicates in three independent assays.

### Statistical analyses

All experiments were performed in three independent tests of triplicates. Statistical analyses were performed with GraphPad Prism 9^®^ software by using Student’s unpaired t test or one-way ANOVA. Statistical significance was set at a p-value of ≤ 0.05.

## Results

### DDB2 expression is reduced in PDAC tumors and correlates with prognosis in PDAC patients

**Figure 1:**
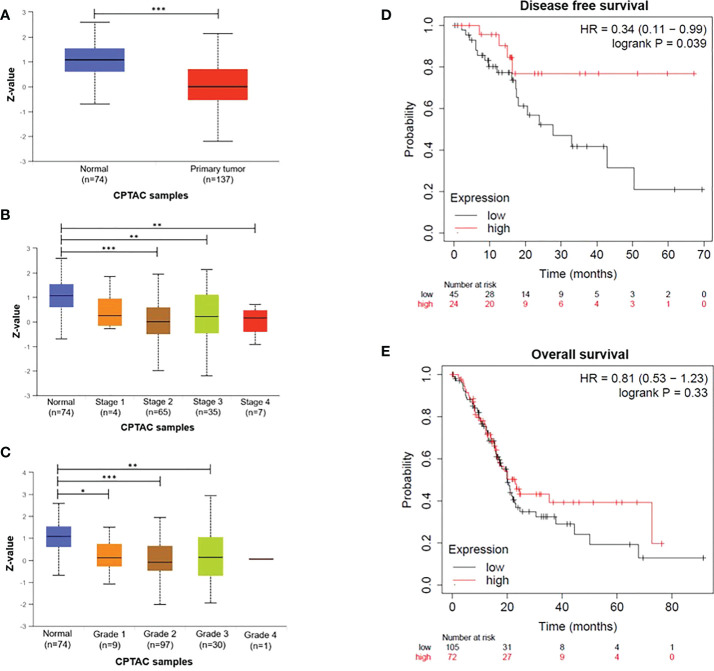
DDB2 expression in PDAC patients **(A)** DDB2 total protein expression in normal tissues (n= 74) and in primary tumors (n= 137), **(B)** Relationship between DDB2 protein expression and tumor stages, **(C)** Relationship between DDB2 protein expression and tumor grades. Data are expressed as mean ± SE, *p < 0.05, **p < 0.01, ***p < 0.001. Protein expression level was expressed as a Z-value, was log2 normalized in each sample, then a Z-value for each sample was calculated as a standard deviation from the median across samples. **(D)** Representative Kaplan-Meier of dis-ease-free survival (DFS) of PDAC patients (n=69) stratified according to DDB2 expression in tumors (log rank p= 0.039) **(E)** Representative Kaplan-Meier of overall survival (OS) of PDAC patients (n=177) stratified according to DDB2 expression in tumors (log rank p= 0.33).

To explore the prognostic value of DDB2 expression in patients with PDAC, we analyzed DDB2 protein expression in PDAC within UALCAN. A reduced proteomic expression was observed in primary tumors compared to normal tissues (*p= 1.02e-10*) (**Figure 1A**). A significant difference in DDB2 protein expression was found between normal tissues and PDAC stages 2, 3 and 4 (*p= 1.24e-8, p= 1.07e-3 and p= 5.51e-3* respectively) as well as tumor grades 1, 2 and 3 (*p= 3.39e-2, p= 5.56e-11 and p= 6.27e-3* respectively) (**Figures 1B, C**). Tumor grade 4 was not evaluated due to the lack of patients in the studied cohort. We did not observe a statistically significant difference in DDB2 RNA expression in the transcriptomic data available (**Supplementary Figure S1**). However, based on survival data analyses using KMplot (www.kmplot.com), low DDB2 mRNA expression was associated with a shorter disease-free survival (DFS) (*p= 0.039*) in PDAC patients (n= 69) but it was not correlated with overall survival (OS) (*p= 0.33*) (n= 177) (**Figures 1D, E**).

### DDB2 is highly expressed in T3M4 cells and poorly expressed in Capan-2 cells

**Figure 2:**
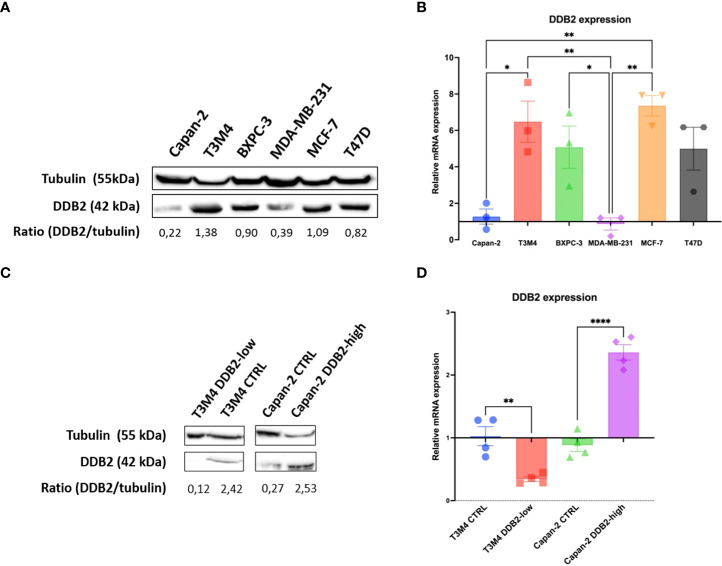
DDB2 expression in PDAC cell lines and in CRISPR-modified cell models. **(A)** DDB2 protein expression evaluated by western blot in three PDAC cell lines (Capan-2, T3M4 and BXPC3) compared to DDB2 high expressing (MCF-7 and T47D) and low expressing (MDA-MB-231) breast cancer cell lines. Tubulin was used as a loading control. **(B)** DDB2 mRNA expression assessed by RT-qPCR. β-actin was used as a housekeeping gene. Results were normalized using h-TERT-HPNE cell line. Data from 3 independent experiments are expressed as mean ± SEM, *p < 0.05, **p < 0.01 (ANOVA). **(C)** DDB2 protein expression determined by western blot in T3M4 CRISPR/Cas9 KO DDB2 cells (T3M4 DDB2-low), Capan-2 CRISPR activation DDB2 cells (Capan-2 DDB2-high) and CRISPR control cells (T3M4 CTRL and Capan-2 CTRL). Tubulin was used as a loading control. **(D)** DDB2 mRNA expression evaluated by RT-qPCR in T3M4 DDB2-low, Capan-2 DDB2-high, T3M4 CTRL and Capan-2 CTRL. β-actin was used as a housekeeping gene. Results were normalized according to DDB2 expression in T3M4 wild type and Capan-2 wild type cells respectively. Data from 3 independent experiments are expressed as mean ± SEM, **p < 0.01, ****p < 0.0001 (Student’s unpaired t test).

DDB2 expression was assessed in three PDAC cell lines (Capan-2, BXPC3 and T3M4) by western blot and RT-qPCR. DDB2 levels were compared with those already described by Kattan et al. in three breast cancer cell lines (DDB2 high levels in T47D and MCF-7, DDB2 low level in MDA-MB-231) (28) (**Figures 2A, B**). DDB2 protein and mRNA were highly expressed in T3M4 and BxPC3 cells while underexpressed in Capan-2 cells. DDB2 expression was reduced in T3M4 cell line, who had the highest expression of DDB2, using a CRISPR/Cas9 KO plasmid and DDB2 was overexpressed in Capan-2 cell line, who had the lowest expression of DDB2, using a CRISPR activation plasmid (**Figures 2C, D**).

### DDB2 has no effect on cell doubling time in T3M4 cell models while DDB2 inhibits the proliferation in Capan-2 cell models

To study the effect of DDB2 on the proliferation of PDAC cell models, we compared cell doubling time of T3M4 DDB2-low and Capan-2 DDB2-high cells with those of wild type and CTRL cells. Any significant difference was noticed between T3M4 DDB2-low cells (24 hours) and T3M4 CTRL (23 hours, *p= 0.3722*). The cell doubling time of Capan-2 DDB2-high cells was significantly increased (86 hours) compared to Capan-2 CTRL (58 hours, *p= 0.0325*) (**Supplementary Figure S2**).

### DDB2 inhibits epithelial-to-mesenchymal transition of the PDAC cell models

**Figure 3:**
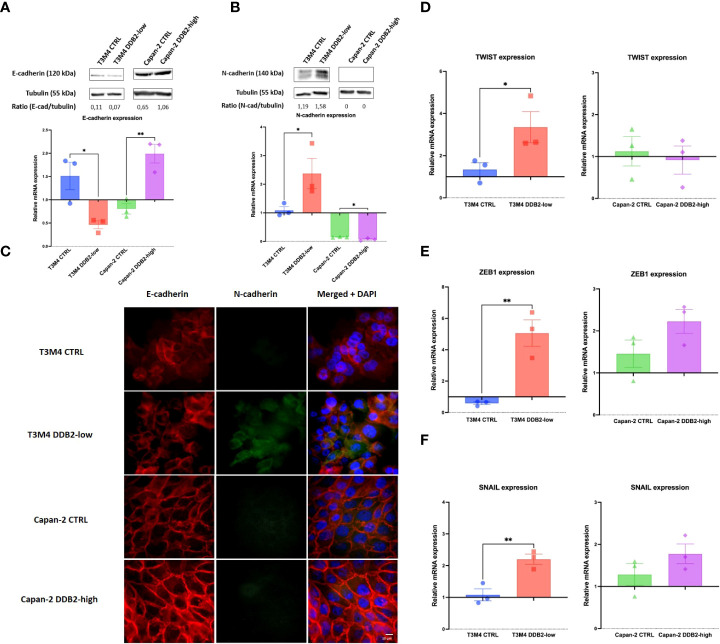
Role of the DDB2 protein in the expression of epithelial-to-mesenchymal transition (EMT) markers in T3M4 DDB2-low, T3M4 CTRL, Capan-2 DDB2-high and Capan-2 CTRL cells. Expression of E-cadherin and N-cadherin were evaluated by **(A, B)** Western blot and RT-qPCR and by **(C)** immunocytochemical analyses. The expression of three EMT transcription factors (EMT-TFs) were studied by RT-qPCR, including **(D)** TWIST, **(E)** ZEB1 and **(F)** SNAIL. β-actin was used as the housekeeping gene, results were normalized according to the expression levels obtained in wild type cells. Data from 3 independent experiments are expressed as mean ± SEM, *p < 0.05, **p < 0.01 (Student’s unpaired t test).

Using phase contrast microscopy, we did not observe any significant morphological shift between control cells and DDB2-modified cells. By immunocytochemical, western blot and RT-qPCR assays, an increase in the expression of the mesenchymal marker N-cadherin and a loss of the epithelial marker E-cadherin were observed in T3M4 DDB2-low cells compared to T3M4 CTRL cells (*p= 0.0389* and *p= 0.0270* respectively) (**Figures 3A–C**). In Capan-2 DDB2-high cells, an increase in E-cadherin protein and mRNA expression (*p= 0.0068*) and a decrease in N-cadherin mRNA level (*p= 0.0332*) were showed while no significant difference in N-cadherin protein expression was noticed (**Figures 3A–C**). We further investigated whether DDB2 can modulate the expression of major EMT-transcription factors (EMT-TFs), including SNAIL, ZEB1 and TWIST. By RT-qPCR, we observed a significant increase in the expression of SNAIL, ZEB1 and TWIST in T3M4 DDB2-low cells compared to T3M4 CTRL cells (*p= 0.0055*; *p= 0.0063* and *p= 0.0335* respectively) (**Figures 3D–F**). The overexpression of DDB2 in Capan-2 DDB2-high cells did not induce any change in the expression of EMT-TFs compared to Capan-2 CTRL cells.

### DDB2 inhibits migration and invasion in PDAC cell models

**Figure 4:**
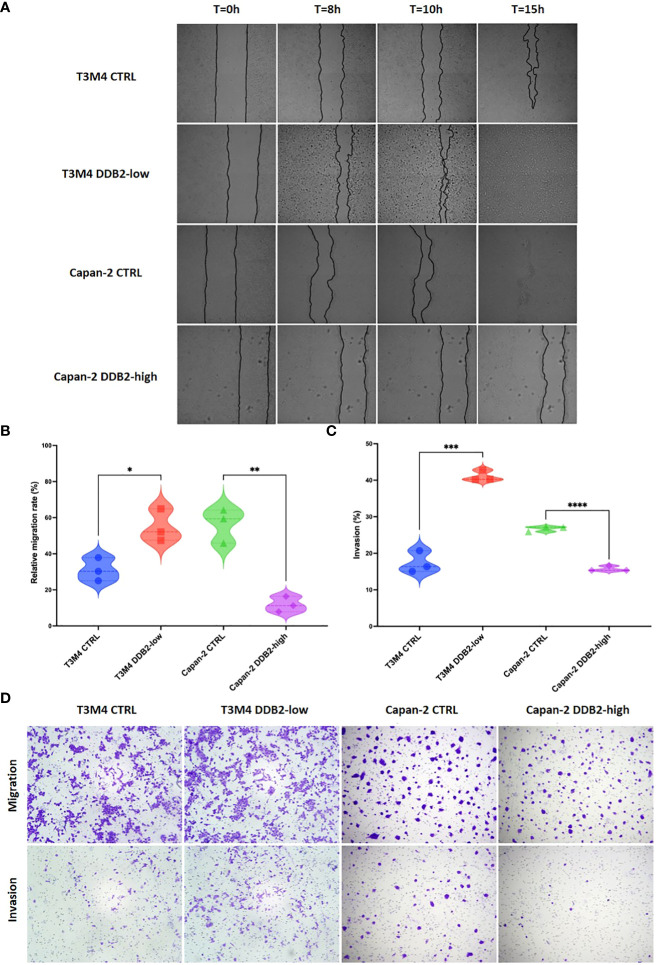
Role of the DDB2 protein in the migration and invasion potential of PDAC cell models. **(A, B)** Analysis of cell migration rate by wound healing assays in T3M4 DDB2-low, T3M4 CTRL, Capan-2 DDB2-high and Capan-2 CTRL cells. Microscopic images of wound healing assays are shown in **(A)** and the quantification of relative migration rate at 8h is shown in **(B)**. Data from 3 independent experiments are expressed as mean ± SEM, *p < 0.05, **p < 0.01 (Student’s unpaired t test). **(C, D)** Analysis of the invasion properties of T3M4 DDB2-low, T3M4 CTRL, Capan-2 DDB2-high and Capan-2 CTRL cells using transwell assays. The quantification of the percentage of invasion is shown in **(C)** and microscopic images are shown in **(D)**. Data from 3 independent experiments are expressed as mean ± SEM, ***p < 0.001, ****p < 0.0001 (Student’s unpaired t test).

To assess the role of DDB2 on the migration properties of PDAC cells, we performed wound healing assays. After 8 hours, the migration rate of T3M4 DDB2-low cells was increased by approximately 23% compared to T3M4 CTRL cells (*p= 0.0207*). Inversely, the migration rate of Capan-2 DDB2-high cells was diminished by more than 44% compared to Capan-2 CTRL cells (*p= 0.0018*) (**Figures 4A, B**). Using transwell assays, T3M4 DDB2-low cells were more invasive than T3M4 CTRL cells (41% vs 17%, *p= 0.0002*) while Capan-2 DDB2-high cells were less invasive than Capan-2 CTRL cells (15% vs 26%, *p< 0.0001*) (**Figures 4C, D**).

### DDB2 increases sensitivity to chemotherapies in Capan-2 cells

**Figure 5:**
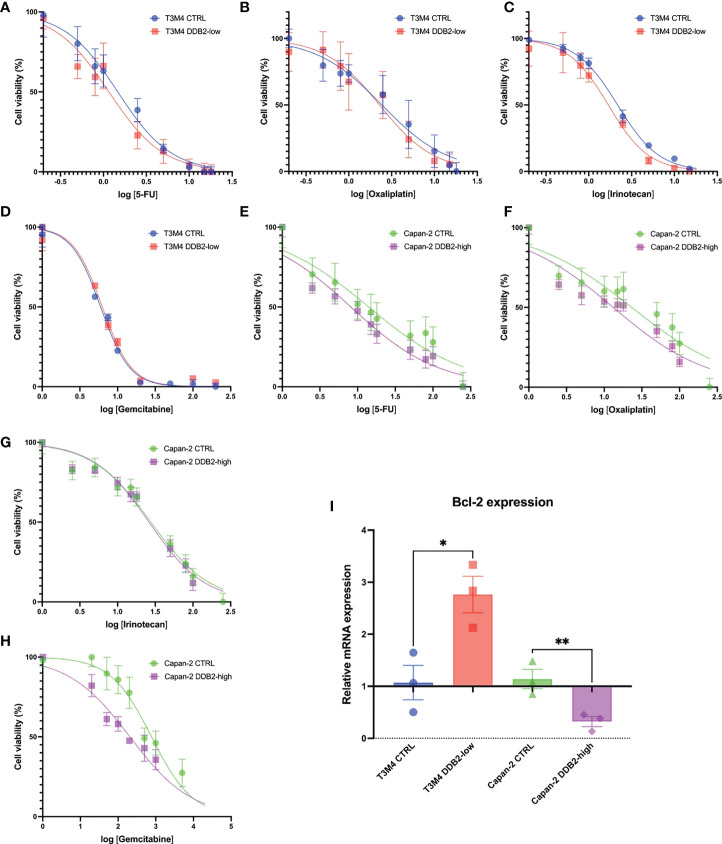
Determination of cell viability of T3M4 CTRL, T3M4 DDB2-low, Capan-2 CTRL and Capan-2 DDB2-high. Cells were treated with increasing concentrations of 5-FU **(A–E)**, oxaliplatin **(B–F)**, irinotecan **(C–G)** and gemcitabine **(D–H)**. Data from 3 independent experiments are expressed as mean ± SEM. **(I)** Expression of Bcl-2 evaluated by RT-qPCR. β-actin was used as the housekeeping gene, results were normalized according to the expression levels obtained in wild type cells. Data from 3 independent experiments are expressed as mean ± SEM, *p < 0.05, **p < 0.01 (Student’s unpaired t test).

We studied the role of DDB2 in the response to 5-fluorouracil (5-FU), oxaliplatin, irinotecan and gemcitabine treatment in our PDAC cell models. The concentration of each chemotherapy which is able to reduce by 50% the cell viability (IC50) was determined. We first observed that the concentrations needed to inhibit cell viability in T3M4 cells were lower than in Capan-2 cells. In T3M4 DDB2-low cells, we did not observe any significant difference in IC50 compared to T3M4 CTRL cells for all the chemotherapeutic agents. The IC50 of 5-FU, oxaliplatin and gemcitabine were lower for Capan-2 DDB2-high than CTRL cells (*p=0.0021; p= 0.0397; p=0.0154* respectively) (**Figure 5** and **Table 2**). We further investigated whether chemotherapy sensitivity was associated with Bcl-2 expression level. By RT-qPCR, we observed a significant increase in Bcl-2 expression in T3M4 DDB2-low cells and a significant decrease in Capan-2 DDB2-high cells (*p=0.0123* and *p=0.0088* respectively) (**Figure 5I**).

**Table 2:** Calculated IC50 of 5-FU, oxaliplatin, irinotecan and gemcitabine for T3M4 DDB2-low, T3M4 CTRL, Capan-2 DDB2-high and Capan-2 CTRL cells.

Cell lines		IC50			
	5-FU (µM)	Oxaliplatin (µM)	Irinotecan (µM)	Gemcitabine (nM)
T3M4 CTRL	2.25 ± 1.28	2.27 ± 2.3	2.37 ± 0.30	6.61 ± 1.14
T3M4 DDB2-low	1.35 ± 0.56	2.36 ± 2.4	1.82 ± 0.42	6.59 ± 0.22
*p*	*0.3267* (NS)	*0.9633* (NS)	*0.1401* (NS)	*0.1441* (NS)
Capan-2 CTRL	15.69 ± 1.14	36.77 ± 12.6	28.74 ± 1.95	728.0 ± 220.5
Capan-2 DDB2-high	7.11 ± 1.77	13.75 ± 4.12	27.10 ± 9.58	190.2 ± 64.65
*p*	*0.0021* (**)	*0.0397* (*)	*0.7862* (NS)	*0.0154* (*)

*p <0.05, **p < 0.01, NS, Not significant.

## Discussion

Here, we showed that high DDB2 levels seem correlated with better prognosis in patients with pancreatic cancer and sensitize PDAC cells to chemotherapy. DDB2 is a potent regulator of EMT and inhibits tumor migration and invasiveness of the studied PDAC cells. Together, our results identify new tumor suppressor functions of DDB2 in pancreatic cancer.

In this study, we reported that DDB2 protein expression level was found lower in PDAC tissues from different stages and grades compared to normal pancreatic tissues. Interestingly we did not find any significant difference in transcriptomic expression of DDB2. These results highlight the interest to focus not only on the genomic landscape but also take into account the proteogenomic approach, that integrates proteomics and post-translational modifications, that might provide a better comprehension of cancer biology (29). We observed that PDAC patients with DDB2-low level had worse DFS. Similar results have been previously described in other cancer models. Notably, a downregulation of DDB2 was already found in advanced oral/head and neck squamous cell carcinoma (HNSCC) and in colon cancer tissues compared to normal tissues and patients with HNSCC harboring low DDB2 mRNA expression level were associated with a shorter overall survival (14, 15).

Previous studies have demonstrated that DDB2 is a potent regulator of EMT in colon adenocarcinoma and in HNSCC (14, 15). In these models, DDB2 has the ability to repress mRNA expression of EMT-TFs SNAIL and ZEB1 and therefore can drive EMT through the expression of epithelial proteins and the repression of mesenchymal proteins (14, 15). In the same way, we identified DDB2 as a repressor of the EMT process in PDAC as DDB2 loss of expression in T3M4 cells enhanced EMT through the activation of EMT-TFs SNAIL, ZEB1 and TWIST while DDB2 overexpression in Capan-2 cells seem to reinforce the epithelial phenotype of the cells by increasing E-cadherin expression. As wild type DDB2-low Capan-2 cells basically harbor an epithelial phenotype, we were not able to evaluate in this model whether the induction of DDB2 expression could reverse EMT and switch cells from mesenchymal to epithelial phenotype as it was previously described in a mesenchymal DDB2-low HNSCC cell model (15).

DDB2 had been already shown as having dual roles in migration and invasion of cancer cells depending on the cancer localization. In breast cancer, DDB2 is overexpressed in non-invasive cells compared to invasive cells and DDB2 re-expression in invasive breast cancer cells limits their motility and invasiveness (28, 30). Inversely, DDB2 has been shown to promote the migration of gastric cancer cells (11). In the two studied PDAC cell models, DDB2 seems to be a negative regulatory factor of migration and invasion, as previously observed in breast cancer. In breast cancer, Ennen et al. found that DDB2 reduces cell invasion by upregulating the cytoplasmic inhibitor IκBα and decreasing NF-κB pathway activity (30). NF-κB pathway is known to be constitutively activated in PDAC, in part due to its activation by KRAS mutation, which is found in almost 90% of PDAC patients (31–33). In this context, it could be of interest to study NF-κB pathway in our PDAC cell models in order to determine whether DDB2 can modulate invasion through the same mechanisms than in breast cancer.

In our study, we evaluated the role of DDB2 in the response of PDAC cell lines to conventional chemotherapies used for the management of patients with PDAC. We first observed that IC50 of 5-FU, oxaliplatin, irinotecan and gemcitabine in DDB2-high T3M4 cells were overall lower than those in DDB2-low Capan-2 cells, suggesting that DDB2 expression might sensitize to chemotherapies. However, Capan-2 cells are known to harbor *TP53* mutations that result in a non-functional p53 protein which could also explain their different response to chemotherapy compared to T3M4 cells that have functional p53. In the same way, overexpression of DDB2 in Capan-2 cells sensitized cells to 5-FU, oxaliplatin and to gemcitabine while no change in IC50 was observed for irinotecan. Previous study demonstrated that overexpression of DDB2 can sensitize ovarian cancer cells to cisplatin treatment through the downregulation of the anti-apoptotic protein Bcl-2 (34). We investigated whether chemotherapy sensitivity was also associated with Bcl-2 expression level in our models. Similarly, we found that DDB2 can downregulate the level of Bcl-2 mRNA. As reported by Barakat et al., Bcl-2 transcription is regulated by the transcription factor E2F1 (35), which itself interacts with DDB2 (36). We can therefore hypothesize that DDB2 can modulate Bcl-2 transcription through the interaction with E2F1. Interestingly, we found an increase in Bcl-2 expression in T3M4 DDB2-low cells that has no effect on chemotherapy sensitivity, the T3M4 cell line being basically sensitive to all chemotherapies tested. Finally, these data suggest that DDB2 could represent a promising prognostic biomarker. To confirm the role of DDB2 in PDAC chemosensitivity, the use of an animal model and a posteriori of a large cohort of patients with PDAC is requested.

Our results suggest that DDB2 influences different steps of PDAC cancerogenesis depending on the PDAC cell model studied. DDB2 deficiency in T3M4 DDB2-low cells was strongly involved in the activation of EMT, migration and invasion while the overexpression of DDB2 in Capan-2 DDB2-high decreased proliferation, migration and invasion and sensitized cells to chemotherapy. T3M4 and Capan-2 cell lines that were used in this study inherently display highly different characteristics in terms of origin, differentiation status, proliferation and migration abilities. Capan-2 cells are derived from primary tumor and are well differentiated whereas T3M4 cells originate from lymph node metastasis and are moderately differentiated. The T3M4 cells are supposed to have metastatic characteristic, and as we have shown in this study, PDAC metastatic cells that have undergone EMT are supposed to have a low DDB2 expression level. However, we found that the T3M4 cell line has a high basal level of DDB2 expression and conversely, the Capan-2 epithelial cell line has a low basal DDB2 expression level. This lack of correlation between basal DDB2 expression level and cell origin was also found in several cell lines from other cancer sites (**Supplementary Table 1**). Once distant, mesenchymal cells can reverse the process through mesenchymal-to-epithelial (MET) allowing them to re-acquire epithelial properties that are crucial to colonize novel sites (37). This may explain the absence of the typical elongated mesenchymal-like morphology and the presence of E-cadherin in the T3M4 cell line. Our results allowed us to conclude on the effect of the change in DDB2 expression in PDAC cells but not on the basal expression level. T3M4 and Capan-2 cell lines also present distinct mutational profiles and gene expression patterns (38, 39). Furthermore, in our models DDB2 is reduced or enhanced but the expression is not strictly knock down which is closer to the reality of tumor heterogeneity in PDAC patients. The singular characteristics and behavior of each PDAC cell line could be explain by the fact that pancreatic cancers are highly heterogeneous at both interpatient and intrapatient levels, highlighting the challenge to identify and develop novel therapeutic strategies (40, 41).

Based on our results, increasing DDB2 expression could improve the prognosis of patients with PDAC, reduce EMT, invasion and migration, and sensitize to chemotherapy. Increasing DDB2 expression might represent a promising new therapeutic strategy but requires a better understanding of the regulatory mechanisms of DDB2. DDB2 has been previously described as transcriptionally activated by the tumor suppressor p53 (42) and mutations in *TP53* gene appear as the second most common mutations found in PDAC (43). Nevertheless, others biological processes seem implied in DDB2 regulation (9) and need to be further explored.

Prior to the identification of the role of DDB2 in cancers, DDB2 has been well described in the NER process and was identified to form an heterodimeric UV-DDB complex with DDB1 protein to initiate DNA repair (8). A previous study investigated the role of DDB1 in PDAC and reported DDB1 as a tumor-promoting factor through the positive regulation of cancer cell proliferation, EMT and chemoresistance. High levels of DDB1 were also correlated with a poor prognosis in PDAC patients (44). In the present study, we found opposite roles of DDB2 in PDAC cells in terms of EMT, migration, invasion, chemosensitivity and prognostic value. Based on these data, UV-DDB seems crucial for NER process but also for cancer development since the two major proteins involved in this complex can be differentially expressed in cancers and might lead to either tumor promoting or tumor suppressing effects. It would be of interest to study with more details the role and impact of UV-DDB and the complex interactions between DDB1 and DDB2 proteins.

To conclude, the present findings bring new tumor suppressor functions of DDB2 in PDAC cell models. DDB2 status in patients with PDAC might represent an interesting prognostic and predictive biomarker.

## Data availability statement

Clinical data used in this article can be found at: www.kmplot.com and at: http://ualcan.path.uab.edu. All other results of the study are available in the article and in the **Supplementary Material**. There is no online repository.

## Author contributions

Conceptualization, JDa, AH, and PG; methodology, JDa, AW, MB, AF, LL, AL, SG, MH, MR, and JDe; data curation, JDa, MM, and LL; formal analysis, JDa, AW, MB, and AF; visualization, AW, AF, AL, SG, MH, MR, and JDe, writing-original draft, JDa and PG; writing-review and editing, JDa, AW, MB, AF, AH, and PG; supervision, J-LM, AH, and PG, funding acquisition, J-LM, AH, and PG, project administration, J-LM, AH, and PG. All authors contributed to the article and approved the submitted version.
