# Supplementary material for: DDB2 represses epithelial-to-mesenchymal transition and sensitizes pancreatic ductal adenocarcinoma cells to chemotherapy
Source: Front Oncol. 2022 Dec 8;12:1052163. doi: 10.3389/fonc.2022.1052163 (PMC9773984; doi:10.3389/fonc.2022.1052163)
Supplement: Supplementary file 1 [file DataSheet_1.docx]

Supplementary Material


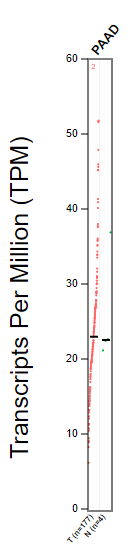


**Supplementary figure S1:** DDB2 RNA expression in PDAC patients. DDB2 mRNA expression in normal tissues (n=4) and in primary tumor (n=179). Data of PAAD cohort from the TCGA obtained with GEPIA webtool.


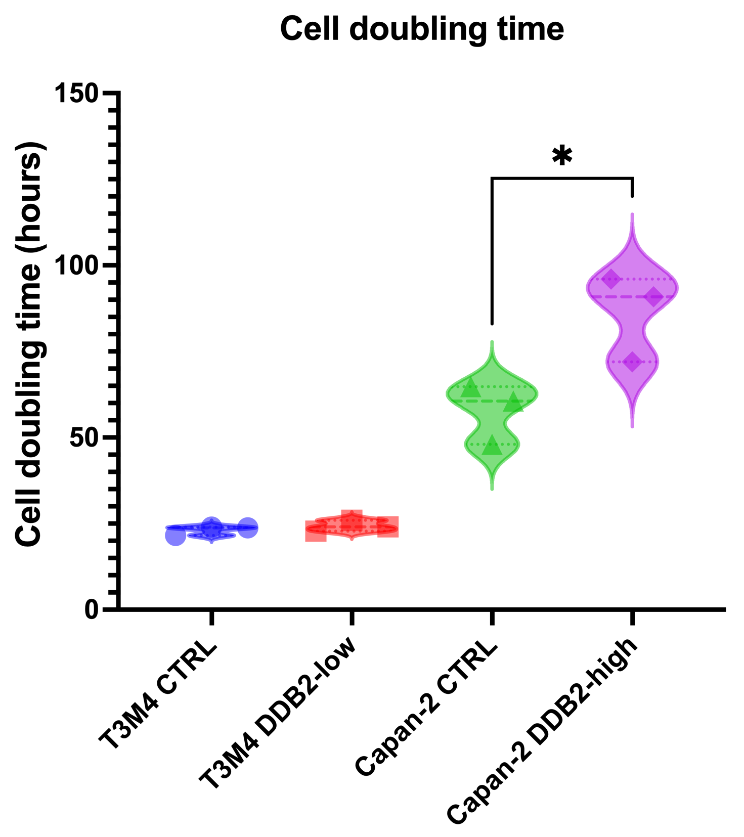


**Supplementary figure S2:** Role of DDB2 in the proliferation of PDAC cell models. Data are represented as cell doubling time of T3M4 CTRL, T3M4 DDB2-low, Capan-2 CTRL and Capan-2 DDB2-high. Data from 3 independent experiments are expressed as mean ± SEM, *p<0.05 (Student’s unpaired t test).

| Cell type | Cell line | Origin | DDB2 status | Reference |
| --- | --- | --- | --- | --- |
| Pancreatic ductal adenocarcinoma (PDAC) | Capan-2 | Primary tumor | Low | Current study |
|  | BXPC3 |  | High |  |
|  | T3M4 | Metastatic site | High |  |
| Breast cancer | SK-BR-3 | Metastatic site | Low | Kattan et al., 2008 |
|  | MDA-MB-231 |  | Low |  |
|  | MCF-7 |  | High |  |
|  | T47D |  | High |  |
| Head and Neck Squamous Cell Carcinoma (HNSCC) | SCC-9 | Primary site | Low | Bommi et al., 2018 |
|  | SCC-4 |  |  |  |
|  | SCC-25 |  |  |  |
|  | SCC-40 |  |  |  |
|  | SCC-15 |  | High |  |
| Ovarian cancer | A2780 | Primary site | High | Barakat et al., 2010 |
|  | CP70 |  | Low |  |
| Colon cancer | SW620 | Metastatic site | Low | Roy et al., 2013 |
|  | SW480 | Primary site | High |  |
|  | HCT116 |  | High |  |

Supplementary table 1: DDB2 expression in cell lines according to their origin.
